# The interplay of fear of pain, emotional states, and pain perception in medical and nursing students: A cross-sectional study

**DOI:** 10.1371/journal.pone.0314094

**Published:** 2024-11-21

**Authors:** João Miguel Romualdo, Elisabete Borges, Isaura Tavares, Daniel H. Pozza

**Affiliations:** 1 Experimental Biology Unit, Department of Biomedicine, Faculty of Medicine of Porto, University of Porto, Porto, Portugal; 2 CINTESIS@RISE, Porto, Portugal; 3 Nursing School of Porto (ESEP), Porto, Portugal; 4 Institute for Research and Innovation in Health and IBMC, University of Porto, Porto, Portugal; Nuclear Science and Technology Research Institute, ISLAMIC REPUBLIC OF IRAN

## Abstract

**Background:**

Fear of pain is known to influence pain perception and worsen pain outcomes. However, its relationship with pain threshold remains unclear. Negative emotional states, namely depression, anxiety, and stress, have been found to increase fear of pain. Previous pain experiences, both undergone and observed, can also influence fear of pain. Furthermore, healthcare students’ interactions with pain patients may be influenced by fear of pain and pain perception. This study aimed to assess fear of pain among Portuguese medical and nursing students, analyse its association with sex, negative emotional states, previous pain experiences, and curricular year, and evaluate the influence of fear of pain on heat pain threshold.

**Methods:**

A survey based on validated Portuguese questionnaires was carried out. Participants were then invited for quantitative sensory tests to assess heat pain threshold.

**Results:**

Fear of pain was similar for medical and nursing students. Female students presented a higher fear of medical pain. Stress was associated with an increased fear of severe pain. Previous painful experiences, particularly those involving medical procedures, have been found to be variably associated with fear of pain, depending on the type and characteristics of these experiences. No associations were found between fear of pain and curricular year, nor between heat pain threshold and sex or fear of pain.

**Conclusions:**

This study highlights relevant aspects of the emotional and cognitive modulation of fear of pain and pain perception in medical and nursing students. The influence of previous pain experiences on fear of pain is also essential because healthcare students are frequently exposed to painful medical situations during their courses. As altered pain perception can influence their role as future healthcare professionals, the study of fear of pain and its modulators in healthcare students represents an important research field.

## Introduction

Pain is a personal experience influenced by biological, psychological, and social factors, learned and shaped through life experiences [1]. It occurs when nociceptors are activated and conduct excitatory signals to the brain. These signals are modulated by numerous factors, with the type and extent of this modulation being thoroughly influenced by previous experiences, expectations, and affective state, resulting in a perception that is extensively influence by various psychosocial factors [2, 3].

While acute pain serves a survival purpose, it can persist beyond the acute event’s resolution, evolving into chronic pain [4], which reduces quality of life and promotes depression and anxiety. However, these negative emotional states (NES) can also facilitate the development of chronic pain [5]. The reciprocal association of chronic pain with mental health issues is a problem which has a massive prevalence in modern societies and worsens pain outcomes [4, 6].

Fear of pain (FOP) is a key psychological factor linked to pain perception. FOP, triggered by an anticipated threat, leads to withdrawal behaviour [7]. Prolonged fear promotes avoidance, which worsens pain and disability [8]. Anxiety and depression can also reinforce FOP [9]. Moreover, FOP can be elicited and indirectly modulated through observation and verbal transmission [10], especially among healthcare students and professionals, who are frequently exposed to patients in pain.

Furthermore, medical and nursing students experience elevated levels of depression, anxiety, and stress [11–13], which may influence their pain perception. However, knowledge about this topic is limited and no research has been performed in Portugal.

Previous pain experiences (PPE) can also influence FOP, with this association being dependant on the exposures considered and the studied population [14].

Additionally, FOP is positively associated with pain intensity [15, 16]. A few studies have also explored the link between FOP and pain threshold, yet findings are inconclusive. Research suggests either a correlation between higher FOP and lower pain threshold [17, 18] or no significant association between these variables [19, 20].

Quantitative sensory tests (QST) are a quick and simple non-invasive method of assessing multiple sensory modalities. Heat pain threshold (HPT) is effective and reliable at experimentally assessing different types of pain [21], constituting a useful test with practical application.

This study aimed to characterize FOP and NES (depression, anxiety, and stress) in Portuguese medical and nursing students and assess their association, in order to evaluate the emotional modulation of pain perception in this population. Secondary objectives included assessing the association between FOP, sex, and PPE and determining whether pain knowledge and clinical exposure to pain (assessed through curricular year) would be linked to lower FOP. Finally, we aimed to determine whether higher FOP would be associated with a lower HPT.

## Materials and methods

This study’s protocol was approved by the Ethical Committee of the Faculty of Medicine of the University of Porto (85/CEFMUP/2022) and the Data Protection Unit of the University of Porto (R-2/2023). An anonymous online survey was carried out among medicine integrated master’s and nursing bachelor’s students. Medical students were from two public medical schools in the north of Portugal. Nursing students were from a public nursing school in the north of Portugal. Submissions from medical students were accepted between March 18^th^ and April 21^st^, 2023. Since the first instance when the survey was shared directly with nursing students was at the end of April, which resulted in a low response rate by April 21^st^, the submission period for these students was extended until May 29^th^, 2023. Current medical and nursing students over 18 years old were included in the study. A few dentistry students also completed the survey. However, the number of responses was so low that no conclusions could have been drawn, so we opted to exclude them.

To assess FOP, the Fear of Pain Questionnaire 9 (FPQ-9) was used. Questions about whether the participants had already experienced each of the painful situations described in the FPQ-9 (or similar) were added, parallel to this questionnaire. The Depression Anxiety and Stress Scale 21 (DASS-21) was used to assess traits of depression, anxiety, and stress. The survey was carried out using validated Portuguese adaptations of the original questionnaires [22, 23]. Questions concerning sociodemographic variables were also included.

As per the originally proposed scoring system for the FPQ-9 [24], questions were categorized into three subscales: Fear of Severe Pain, Fear of Minor Pain, and Fear of Medical/Dental Pain. Each item was rated on an ordinal scale ranging from 1 to 5. The score for Fear of Severe Pain was determined by summing the values for questions 1, 6, and 9. The score for Fear of Minor Pain was determined by summing the values for questions 3, 5, and 7. The score for Fear of Medical Pain was determined by summing the values for questions 2, 4, and 8. Finally, the Total Fear of Pain score was obtained by summing the values for all nine items [24].

The DASS-21 was scored according to the originally proposed approach, which involved dividing the questionnaire into three scales: Depression, Anxiety, and Stress [25]. Each item was rated on an ordinal scale ranging from 0 to 3. The Depression score was obtained by summing the values for questions 3, 5, 10, 13, 16, 17, and 21. The Anxiety score was obtained by summing the values for questions 2, 4, 7, 9, 15, 19, and 20. The Stress score was obtained by summing the values for questions 1, 6, 8, 11, 12, 14, and 18. Subsequently, these values were converted to severity ratings (Normal, Mild, Moderate, Severe, Very Severe), according to the questionnaire’s scoring guide [26].

Following the survey’s submission, participants were invited to volunteer for follow-up QST. Only individuals without prior or current history of chronic pain were considered for this assessment. These tests took place in the Faculty of Medicine of the University of Porto between April 10^th^ and June 2^nd^, 2023.

Based on current literature [21, 27–32], all QST were held in a closed-door air-conditioned room with a controlled temperature of 23°C (± 1°C) and were carried out by a single previously trained researcher. The training involved several sessions using the equipment, initially without volunteers and then with individuals other than those included in the results of this research, until a high level of comfort in conducting the tests was achieved. After the calibration of the equipment by the senior researcher, who has 15 years of experience with this equipment and verified it without the volunteers, a 30 x 30 mm Medoc TSA II thermode, was carefully attached to the medial-ventral surface of the volunteer’s non-dominant forearm to provide the thermal stimuli. Participants were asked to confirm that they were thermally comfortable and felt no pain before initiating the test. Before beginning the QST, participants received a detailed explanation of the test and were encouraged to voice any questions or concerns they might have had. The potential influence of environmental factors was minimized since the temperature was kept constant, all electronic devices with notifications were required to be turned off, and no noise could be heard in the testing room. Furthermore, HPT is a consistent test with demonstrated reliability and repeatability [31].

A baseline temperature of 32°C was gradually increased at a rate of 1°C/s, with a maximum threshold of 50°C. Volunteers were instructed to press a button to interrupt the thermal stimulus as soon as it transitioned from a non-painful warmth to an uncomfortable or painful sensation. The specific criteria used to define the heat pain threshold involved registering the temperature on the TSA II program when the button was pressed and subsequently decreasing the device’s temperature at a rate of 8°C/s. Six measurements were conducted, with the mean of the last three stimuli determining the HPT.

During the initial explanation, it was emphasised that participants should not endure the uncomfortable/painful stimuli, as it was not a resistance/tolerance test. Additionally, the computer screen with the TSA II programme was not visible to the participants to avoid influencing their perception. Participants were unaware of the study’s hypothesis.

Before the performance of the QST, participants also signed an informed consent form and completed the FPQ-9 and PPE questions once again, to account for variations in FOP and PPE since answering the previous online survey.

### Statistical analysis

Statistical analyses were carried out using IBM SPSS Statistics version 28. Absolute and relative frequencies were used to describe categorical variables. The data were assessed for normal distribution using the Kolmogorov-Smirnov and Shapiro-Wilk tests, along with visual inspection of histograms. Since all studied variables showed a non-normal distribution, the Mann-Whitney U test and Spearman correlation coefficient were applied. To compare categorical variables, the Chi-square test was used, and when appropriate, the Fisher-Freeman-Halton exact test was conducted. The statistical significance of the tests was assessed at a *p*-value lower than 0.05 (95% confidence interval). Pairwise deletion was performed for missing data. For the FPQ-9, if a participant’s response to any question was missing, they were treated as a missing case for both the relevant subscale and the Total Fear of Pain scale and were therefore excluded from analyses involving those variables. Similarly, for the DASS-21, if a participant’s response to a question was missing, they were considered a missing case for the corresponding subscale. However, for both scales, participants were not excluded from analyses that did not involve the missing data (i.e., other subscales).

## Results

A total of 625 questionnaires were submitted to the study. 42 responses were excluded due to not meeting the inclusion criteria. Exclusions related to area of study encompassed: 16 dentistry students, 2 students did not indicate their area of study, 2 veterinary medicine students, 2 nursing master’s students, 1 nursing PhD student, and 1 health education master’s student. Other reasons for exclusions involved: 16 students from schools outside the three institutions considered and 2 students under 18 years old.

Regarding missing data, there was 1 missing case for curricular year (1 nursing student), 44 missing cases for Fear of Severe Pain (27 medical students, 17 nursing students), 54 missing cases for Fear of Minor Pain (33 medical students, 21 nursing students), 46 missing cases for Fear of Medical Pain (27 medical students, 19 nursing students), 77 missing cases for Total Fear of Pain (50 medical students, 27 nursing students), 11 missing cases for Depression Severity Rating (8 medical students, 3 nursing students), 6 missing cases for Anxiety Severity Rating (5 medical students, 1 nursing student), and 9 missing cases for Stress Severity Rating (8 medical students, 1 nursing student). For all other variables, there were no missing cases.

To calculate the sample size, a population of 446,000 students in Portugal (across all areas of knowledge, not just health) was considered at the time of the study. A minimum of 384 participants would be required to achieve a 5% margin of error. A convenience sampling method was employed to include more than 384 participants. To ensure adequate statistical power in the analysis, a power calculation was performed. Based on a standard deviation of 2.22 for medical students, 1.48 for nursing students, and a pooled standard deviation of 1.89, we assumed a small effect size (Cohen’s d = 0.2) and a significance level of 0.05. The analysis revealed that a sample size of approximately 384 participants would be required to achieve 80% power. With a total of 583 participants, the study exceeds the required sample size, providing a power of 92.7%, indicating sufficient sensitivity to detect small effects. However, none of the studied variables followed a normal distribution (p < 0.001, Kolmogorov-Smirnov and Shapiro-Wilk tests); thus, nonparametric tests were applied.

Of the 583 participants included in the study, the median age was 20 years old (ranging from 18 to 45); 78.4% were female and 21.4% male; 28.0% were nursing bachelor’s students and 72.0% medicine integrated master’s students. Participants’ detailed demographic characteristics, FPQ-9 scores, and DASS-21 severity ratings are presented in Table 1.

**Table 1:** Participants’ demographic characteristics, FPQ-9 scores, and DASS-21 severity ratings for the total study sample and the medical and nursing students subgroups.

	Survey Participants (N = 583)	Medical students (N = 420)	Nursing students (N = 163)	*p*-value
**Sex, n (%)**				
Female	457 (78.4%)	315 (75.0%)	142 (87.1%)	0.002*
Male	125 (21.4%)	104 (24.8%)	21 (12.9%)	
Other	1 (0.2%)	1 (0.2%)	0 (0.0%)	
**Age, median** **[interquartile range]**	20 [19.0, 22.0]	20.0 [19.0, 22.0]	20.0 [19.0, 21.5]	0.667†
**Curricular year, n (%)**				
1	—	105 (25.0%)	20 (12.3%)	— ^a^
2	—	120 (28.6%)	74 (45.4%)	
3	—	45 (10.7%)	41 (25.2%)	
4	—	48 (11.4%)	27 (16.6%)	
5	—	67 (16.0%)	—	
6	—	35 (8.3%)	—	
**FPQ-9, median** **[interquartile range]**				
Fear of Severe Pain	11 [10, 13]	11 [10, 13]	11 [10, 12]	0.491†
Fear of Minor Pain	6 [5, 8]	6 [5, 8]	6 [5, 8]	0.267†
Fear of Medical Pain	8 [6,99]	8 [6, 9]	8 [6, 9]	0.826†
Total Fear of Pain	25.00 [21.8, 29.0]	25.00 [21.8, 29.0]	25.00 [21.3, 29.0]	0.777†
**DASS-21**				
**Depression Severity, n (%)**				
Normal	425 (74.3%)	308 (74.8%)	117 (73.1%)	0.507†
Mild	46 (8.0%)	36 (8.7%)	10 (6.3%)	
Moderate	59 (10.3%)	42 (10.2%)	17 (10.6%)	
Severe	23 (4.0%)	14 (3.4%)	9 (5.6%)	
Extremely Severe	19 (3.3%)	12 (2.9%)	7 (4.4%)	
**Anxiety Severity, n (%)**				
Normal	444 (76.9%)	326 (78.6%)	118 (72.8%)	0.065†
Mild	45 (7.8%)	35 (8.4%)	10 (6.2%)	
Moderate	49 (8.5%)	34 (8.2%)	15 (9.3%)	
Severe	32 (5.5%)	18 (4.3%)	14 (8.6%)	
Extremely Severe	7 (1.2%)	2 (0.5%)	5 (3.1%)	
**Stress Severity, n (%)**				
Normal	289 (50.3%)	207 (50.2%)	82 (50.6%)	0.737†
Mild	93 (16.2%)	69 (16.7%)	24 (14.8%)	
Moderate	87 (15.2%)	64 (15.5%)	23 (14.2%)	
Severe	73 (12.7%)	53 (12.9%)	20 (12.3%)	
Extremely Severe	32 (5.6%)	19 (4.6%)	13 (8.0%)	

*Chi-square or Fisher-Freeman-Halton exact test.

†Mann-Whitney U test.

^a^A comparative analysis was not performed for Curricular year as this variable was deemed not comparable between the two areas of study.

Descriptive and comparative analyses regarding medical and nursing students subgroups were also carried out (Table 1). There was a higher percentage of female students in the nursing students subgroup (*p* = 0.002). Age, FPQ-9 scores, and DASS-21 severity ratings were similar between the two subgroups.

### Association of sex and DASS-21 severity ratings with FOP

Table 2 presents the analyses conducted regarding the association of sex, depression severity rating, anxiety severity rating, and stress severity rating with FOP.

**Table 2:** Association/correlation of sex and DASS-21 severity ratings with fear of pain.

	Total Sample				Medical Students				Nursing Students			
	Fear of Severe Pain	Fear of Minor Pain	Fear of Medical Pain	Total Fear of Pain	Fear of Severe Pain	Fear of Minor Pain	Fear of Medical Pain	Total Fear of Pain	Fear of Severe Pain	Fear of Minor Pain	Fear of Medical Pain	Total Fear of Pain
**Sex,** ***p*-value** †	0.242	0.246	**0.021**	0.378	0.406	**0.025**	0.125	0.968	0.211	0.105	**0.025**	0.053
**Depression Severity,** ***p*-value** **(*r*-value)** ‡	0.338	0.149	0.108	0.117	0.252	0.042	0.179	0.062	0.804	0.179	**0.016**	0.144
	(0.042)	(0.063)	(0.070)	(0.070)	(0.058)	**(0.104)**	(0.069)	(0.098)	(0.021)	(0.115)	**(0.202)**	(0.127)
**Anxiety Severity,** ***p*-value** **(*r*-value)** ‡	0.117	0.107	0.321	0.223	**0.047**	0.085	0.155	**0.045**	0.677	0.260	0.064	0.418
	(0.068)	(0.070)	(0.043)	(0.055)	**(0.101)**	(0.088)	(0.072)	**(0.105)**	(0.035)	(0.096)	(0.155)	(0.070)
**Stress Severity,** ***p*-value** **(*r*-value)** ‡	**0.038**	0.117	0.132	0.073	0.077	0.102	0.403	0.122	0.314	0.743	**0.023**	0.224
	**(0.090)**	(0.069)	(0.066)	(0.080)	(0.090)	(0.084)	(0.043)	(0.584)	(0.084)	(0.028)	**(0.190)**	(0.105)

Legend: †Mann-Whitney U test.

‡ Spearman correlation coefficient.

For the total sample, an association was found between Fear of Medical Pain and Sex (*p* = 0.021), with a median Fear of Medical Pain of 8 for females and 7 for males. A significant correlation was also found between Fear of Severe Pain and Stress Severity (*r* = 0.090, *p* = 0.038).

Subgroup analyses were also performed for the two areas of study (Table 2).

Regarding the medical students subgroup, an association was found between Fear of Minor Pain and Sex (*p* = 0.025), with a median Fear of Minor Pain of 6 for females and 7 for males. Significant correlations were also found between Fear of Severe Pain and Anxiety Severity (*r* = 0.101, *p* = 0.047) and between Fear of Total Pain and Anxiety Severity (*r* = 0.105, p = 0.045).

Regarding the nursing students subgroup, an association was found between Fear of Medical Pain and Sex (*p* = 0.025), with a median Fear of Medical Pain of 8 for females and 6 for males. Significant correlations were also found between Fear of Medical Pain and Depression Severity (*r* = 0.202, *p* = 0.016) and between Fear of Medical Pain and Stress Severity (*r* = 0.190, *p* = 0.023).

### Association of PPE with FOP

Two separate analyses were carried out to assess the association between PPE and FOP.

Firstly, an analysis was performed to assess the association between having previously experienced a specific situation described in the FPQ-9 questions and fear of that particular pain. Results are presented in Table 3.

**Table 3:** Association between a previous experience of a specific painful situation and fear of that particular pain.

	Previous pain experience for each FPQ-9 question^a^
**Fear of Pain, *p*-value** ^b^	
Fear of Pain for question 1	0.455
Fear of Pain for question 2	0.446
Fear of Pain for question 3	0.110
Fear of Pain for question 4	**0.032**
Fear of Pain for question 5	0.061
Fear of Pain for question 6	0.101
Fear of Pain for question 7	0.304
Fear of Pain for question 8	0.980
Fear of Pain for question 9	**0.003**

^a^e.g.: “Previous pain experience for question 1” for the variable “Fear of Pain for question 1”, “Previous pain experience for question 2” for the variable “Fear of Pain for question 2”, etc.

^b^The Mann-Whitney U test was performed for all analyses.

A PPE of “Receiving an injection in your mouth” (question 4) was associated with a lower FOP for question 4 (*p* = 0.032), with a median of 2 for individuals with this PPE and 3 for individuals without this PPE. A PPE of “Falling down a flight of concrete stairs” (question 9) was associated with a lower FOP for question 9 (*p* = 0.003), with a median of 3 for individuals with this PPE and 4 for individuals without this PPE.

Secondly, another analysis was carried out to assess the correlation between having previously experienced multiple of the different painful situations described in the questions from each FPQ-9 subscale (Severe Pain, Minor Pain, and Medical Pain) and FOP for the corresponding FPQ-9 subscale. Results can be found in Table 4.

**Table 4:** Correlation between previous experiences of different groups of painful situations and fear of pain.

	Previous experiences of different groups of painful situations (FPQ-9 subscales)^a^
**Fear of Pain, *p*-value (*r*-value)** ^b^	
Fear of Severe Pain	0.688 (-0.017)^c^
Fear of Minor Pain	0.876 (0.007)^d^
Fear of Medical Pain	**0.001 (0.138)** ^e^

^a^e.g.: “Previous experiences of severe pain” for the variable “Fear of Severe Pain”.

^b^The Spearman correlation coefficient was performed for all analyses.

^c^Severe pain questions: questions 1, 6 and 9.

^d^Minor pain questions: questions 3, 5 and 7.

^e^Medical pain questions: questions 2, 4 and 8.

PPE in various situations described in the questions on the Fear of Medical Pain subscale (“Having a foot doctor remove a wart from your foot with a sharp instrument”, “Receiving an injection in your mouth”, and “Receiving an injection in your hip/buttocks) were positively correlated with Fear of Medical Pain (*p* = 0.001, *r* = 0.138).

### Association of curricular year with FOP

Correlation analyses (using the Spearman correlation coefficient) were performed to assess the association between the curricular year in which students were enrolled and FOP. For medical students, no correlations were found for curricular year and Fear of Severe Pain (*p* = 0.332, *r* = -0.049), Fear of Minor Pain (*p* = 0.737, *r* = 0.017), Fear of Medical Pain (*p* = 0.077, *r* = -0.089), or Total Fear of Pain (*p* = 0.607, *r* = -0.027). For nursing students, no correlations were found for curricular year and Fear of Severe Pain (*p* = 0.886, *r* = -0.012), Fear of Minor Pain (*p* = 0.667, *r* = 0.037), Fear of Medical Pain (*p* = 0.859, *r* = -0.015), or Total Fear of Pain (*p* = 0.710, *r* = 0.032).

### Association of FOP with HPT

106 of the 583 participants included in the study volunteered to undergo HPT testing. 87.7% of them were medical students and 12.3% nursing students. The volunteers’ sex distribution (83.0% female, 17.0% male) was similar to that of the total survey sample. However, these volunteers were older (*p*<0.001; median age: 23, interquartile range: [20, 23]), and medical students were enrolled in higher curricular years (*p*<0.001; 14.0% 1^st^ year students, 21.5% 2^nd^ year students, 7.5% 3^rd^ year students, 18.3% 4^th^ year students, 33.3% 5^th^ year students, and 5.4% 6^th^ year students).

The median HPT for this sample was 46.8°C, with an interquartile range of [44.6, 48.2] and results ranging from 38.6°C to 50.0°C.

Statistical analyses were carried out to assess the association of HPT with FOP, sex, age, area of study, and curricular year. Results can be found in Table 5.

**Table 5:** Association/correlation of heat pain threshold with participants’ characteristics and fear of pain scores.

	Heat pain threshold	*p*-value
**Sex, median [interquartile range]**		
Female	46.9 [44.6, 48.3]	0.659†
Male	45.9 [44.5, 48.2]	
**Age**	—	0.244 (r = -0.114)‡
**Area of study, median [interquartile range]**		
Medicine	46.833 [44.4, 48.3]	0.616†
Nursing	46.933 [45.2, 48.2]	
**Fear of Severe Pain**	—	0.185 (r = -0.133)‡
**Fear of Minor Pain**	—	0.855 (r = 0.018)‡
**Fear of Medical Pain**	—	0.242 (r = -0.117)‡
**Total Fear of Pain**	—	0.614 (r = -0.052)‡
**Medical students subgroup** ^a^		
**Curricular year, median [interquartile range]**		
1	43.600 [40.4, 47.7]	0.589 (r = -0.053)‡
2	48.233 [46.9, 48.9]	
3	48.067 [45.7, 50.0]	
4	46.733 [45.3, 48.0]	
5	45.867 [43.2, 47.9]	
6	45.533 [43.6, 47.4]	

†Mann-Whitney U test.

‡Spearman correlation coefficient.

^a^The analysis for Curricular year was limited to medical students, as nursing students were deemed not comparable to medical students in this regard.

HPT was not associated with Total Fear of Pain (*p* = 0.614, *r* = -0.052) or any FPQ-9 subscale.

## Discussion

To the best of our knowledge, this study represents the first investigation on the association of FOP with NES (depression, anxiety, stress), sex, PPE, and HPT among Portuguese medical and nursing students.

FOP was similar for both medical and nursing students, an interesting finding considering the different structures of these courses. Previous studies have found similar FOP scores among peers of the same age [33], suggesting that these students’ FOP is similar to that of the general population. Our results differed from another survey performed in Poland, which suggested that healthcare students had a lower FOP [34], but were similar to those form a different study performed in Turkey [35]. Although cultural, ethnical and racial differences can influence FOP and pain perception [36, 37], which may explain this variability, future studies could benefit from comparing these diverse groups of students. Such research could explore differences in course structures, particularly regarding students’ exposure to patients in pain and the content of pain-related classes in Portugal and Poland, as these factors may also contribute to the observed findings.

Female students presented an increased fear of medical pain, a result consistent with previous research [34]. Women’s responses to the FPQ may be influenced by their interpretation of the consequences of the experiences described [17], which could have reinforced the association found. Female students may express a greater fear not only of medical pain, but also of the repercussions of medical experiences. On the contrary, no association was found between sex and HPT. Previous research has suggested that women have a lower pain threshold [17, 38]. However, this difference has been found to be largely explained by gender role expectations and willingness to report pain [7, 39]. As gender role expectations are becoming less pervasive in younger generations [40], the participants’ overall younger age could have justified a decreased influence of these confounders, further supporting the validity of our findings.

Stress was associated with a heightened fear of severe pain, a result that, to the best of our knowledge, has not previously been described. The strength of this association may have been reinforced by the fact that stress can intensify concerns about the consequences of serious incidents, especially among females [17].

Depression was associated with an increased fear of medical pain, but only among nursing students, which is puzzling. Since the nursing students subgroup had a higher proportion of females and this sex was associated with a higher fear of medical pain, this imbalance could have contributed to the observed finding. However, this aspect should be elucidated in future studies both through the inclusion of more male nursing students and through a more in-depth analysis of these students’ emotional profile. Similarly, male sex and anxiety were linked to a heightened fear of minor pain, but only among medical students. The fact that men are typically considered to underreported pain [41] could justify why the former association has not been described previously. However, since these associations were exclusive to medical students, increasing the number of nursing students enrolled may enable the clarification of these results.

Concerning the relationship between PPE and FOP, a previous experience of falling down a flight of concrete stairs was associated with a reduced fear of that pain. This could be justified by a reduction of both fear of the pain itself and fear of the consequences of such an accident. Women’s higher fear of the consequences of serious incidents [17] might also have reinforced this association, given the high proportion of females in the study’s sample.

A previous experience of receiving an injection in the mouth (a question from the Fear of Medical Pain subscale) was also linked to a lower fear of that pain, consistent with previous studies [14, 42]. On the contrary, when previous painful experiences of the situations described in the Fear of Medical Pain subscale were considered collectively, they were associated with a higher fear of medical pain. This result was unexpected, as it diverged from both the association found for a PPE of receiving an injection in the mouth and the results of a previous study [14]. However, since recurrent and first-time PPE have been found to be differently associated with FOP [8, 34], exposure to multiple painful medical experiences, especially of different types, may affect FOP differently than the experience of a single specific situation. As we did not quantify the number of times each painful situation was experienced, a more in-depth characterization of PPE by ongoing studies could help clarify these conclusions.

Furthermore, the absence of other PPE associations highlights the variability in the relationship between PPE and FOP, depending on the context and characteristics of the experience. Characterizing pain severity for each exposure, lesion resolution (complete or with sequalae) and the necessity of medical and/or surgical care could also help characterize the PPE-FOP relationship further.

The lack of association found between curricular year and FOP contrasted with our expectations, given that Polish medical students in higher curricular years have previously been found to report lower levels of fear of medical pain [34]. Given that the curricular year may indirectly reflect pain knowledge and clinical exposure, and that pain neuroscience education has previously been associated with lower FOP [43, 44], the absence of association found may be attributable to a limited pain curriculum. As insufficient pain education has been previously reported for medical students [45, 46], future studies should aim to investigate whether improving pain knowledge can reduce FOP, which may also help clarify the implications of this result.

Concerning NES, their prevalence was found to be similar in medical and nursing students. The DASS-21 indicated rates of 25.6% for any level of depression, 23.0% for any level of anxiety, and 49.7% for any level of stress. However, considering that the DASS stress scale is described as measuring a “nervous tension, similar to the diagnosis of Generalized Anxiety Disorder” [26], the overall prevalence of anxious symptoms might be even higher. Nonetheless, a recent study on Portuguese medical students reported a high prevalence of NES, with comparable rates of anxiety and depression [11].

The lack of associations found in the QST suggests that FOP does not significantly influence the HPT in the studied non-chronic pain population, according to the protocol utilized. This finding raises important implications for understanding the relationship between psychological factors and pain perception. One potential reason for this lack of association could be that the mild discomfort or very mild pain sensation elicited at the studied threshold does not accurately reflect the complex nature of actual painful experiences. As a result, the HPT may not serve as a reliable measure of pain perception in contexts similar to our investigation, where the nuances of fear and pain are not fully captured. Future research should explore this relationship further, considering different methodologies or participant populations that might yield more significant insights into how fear of pain interacts with pain thresholds. Additionally, examining alternative measures of pain perception or conducting a comprehensive psychological analysis could provide a clearer understanding of the dynamics between FOP and pain experiences.

Regarding missing data, pairwise deletion was employed to maximize the amount of data included in each analysis. This method is not expected to have influenced the results, as the missing data is believed to be completely at random, with no significant differences found between participants with and without missing data. There were 7.5% missing cases for Fear of Severe Pain, 9.3% for Fear of Minor Pain, 7.9% for Fear of Medical Pain, and 13.2% for Total Fear of Pain. Since this data is assumed to be missing at random, with no specific trends identified among participants who did not answer certain questions, the effect of missing cases on the FPQ-9 is expected to be minimal. Moreover, since Total Fear of Pain was the only variable with a missing percentage above the data bias threshold of 10% [47], the influence of missing data on these results is anticipated to be minimal. However, the possibility that these missing cases corresponded to a group of students with a shared characteristic not measured in the study cannot be excluded. Although it is very unlikely, this could have induced bias in the results regarding the FPQ-9 and its associations. For the DASS-21, there were 1.9% missing cases for Depression Severity Rating, 1.0% for Anxiety Severity Rating, and 1.5% for Stress Severity Rating. Therefore, as this proportion of missing cases is not expected to cause data bias, it is very unlikely that the reliability of the results concerning the DASS-21 was affected.

Among the study’s limitations is its cross-sectional design, which prevents us from establishing causal relationships between the studied variables. Additionally, the reliance on self-reported data, and not having clinical assessments, introduces potential biases, such as social desirability bias and recall bias, which may affect the accuracy of participants’ responses. Furthermore, as all participants were students from schools in the north of Portugal, results cannot be extrapolated to other populations. The conclusions’ validity may also be limited for males, as the study’s sample was comprised predominantly of females. This was expected, as a high female proportion is a feature of medical and nursing courses in Portugal. However, the exclusive use of validated Portuguese adaptations of the FPQ-9 and DASS-21 assured the variables measured were the intended ones and the use of their short versions reduced the likelihood of participant fatigue, further improving internal validity. Future longitudinal or experimental studies are necessary to confirm these relationships and reduce the influence of such biases.

## Conclusions

The emotional and cognitive modulation of pain is essential to its perception, making FOP an important variable to consider when assessing pain. Despite the distinct structures of their courses, medical and nursing students exhibit similar FOP. The varying influence of PPE (especially previous medical experiences) on FOP makes it a very relevant matter, especially considering that FOP can be modulated by the observation of pain experiences in others [10] and that medical and nursing students frequently contact with painful medical conditions and procedures. Future studies should aim to quantify the observation of painful medical experiences by medical and nursing students in their interactions with patients and assess the association of this variable both with their FOP and their ability to interact with patients in pain.

Therefore, whether FOP (and pain perception in general) may influence the future activity of these students as healthcare professionals is a question that must be contemplated and raises the need for a deeper understanding of their emotions and cognitions in the context of pain. The influence of the studied variables on FOP, along with the best ways to modulate them, may represent an important field for future research.

A deeper understanding of the emotional and cognitive variables behind the modulation of FOP can assist in the development of targeted interventions to address pain perception in healthcare students and promote better interactions with patients in pain during clinical practice. Furthermore, clarifying the role of PPE (both experienced and observed) and pain knowledge in the modulation of FOP may promote a tailored approach to the education and preparation of these students for their clinical interaction with pain. Previous research has shown that the FOP and pain beliefs of healthcare students affect their approach to pain and pain management [48].

Clarifying these topics should enable a better capacitation of future doctors and nurses for their contact with pain patients, with the ultimate goal of improving pain outcomes.

## Supporting information

S1 Dataset Online survey dataset. Dataset from the online survey.
(XLSX)

S2 Dataset Quantitative sensory tests dataset. Dataset from the follow-up quantitative sensory tests (incuding the recompleted questionnaire).
(XLSX)
